# Polypeptide Substrate Accessibility Hypothesis: Gain-of-Function R206H Mutation Allosterically Affects Activin Receptor-like Protein Kinase Activity

**DOI:** 10.3390/biom13071129

**Published:** 2023-07-14

**Authors:** Jay C. Groppe, Guorong Lu, Mary R. Tandang-Silvas, Anupama Pathi, Shruti Konda, Jingfeng Wu, Viet Q. Le, Andria L. Culbert, Eileen M. Shore, Kristi A. Wharton, Frederick S. Kaplan

**Affiliations:** 1 Department of Biomedical Sciences, Texas A&M University College of Dentistry, 3302 Gaston Ave, Dallas, TX 75246, USA; 2 Department of Molecular Biology, Cell Biology and Biochemistry, Brown University, Providence, RI 02912, USA; 3 Program in Molecular Medicine, Boston Children’s Hospital, Boston, MA 02115, USA; 4 Department of Biological Chemistry and Molecular Pharmacology, Harvard Medical School, Boston, MA 02115, USA; 5 Department of Orthopaedics, University of Pennsylvania School of Medicine, Philadelphia, PA 19104, USA

**Keywords:** allosteric regulation, bone morphogenetic protein (BMP), serine/threonine kinase, ACVR1, ALK2, FKBP12, BMPRII, Smad MH2

## Abstract

Although structurally similar to type II counterparts, type I or activin receptor-like kinases (ALKs) are set apart by a metastable helix–loop–helix (HLH) element preceding the protein kinase domain that, according to a longstanding paradigm, serves passive albeit critical roles as an inhibitor-to-substrate-binding switch. A single recurrent mutation in the codon of the penultimate residue, directly adjacent the position of a constitutively activating substitution, causes milder activation of ACVR1/ALK2 leading to sporadic heterotopic bone deposition in patients presenting with fibrodysplasia ossificans progressiva, or FOP. To determine the protein structural–functional basis for the gain of function, R206H mutant, Q207D (aspartate-substituted caALK2) and HLH subdomain-truncated (208 Ntrunc) forms were compared to one another and the wild-type enzyme through in vitro kinase and protein–protein interaction analyses that were complemented by signaling read-out (p-Smad) in primary mouse embryonic fibroblasts and *Drosophila* S2 cells. Contrary to the paradigm, the HLH subdomain actively suppressed the phosphotransferase activity of the enzyme, even in the absence of FKBP12. Unexpectedly, perturbation of the HLH subdomain elevated kinase activity at a distance, i.e., allosterically, at the ATP-binding and polypeptide-interacting active site cleft. Accessibility to polypeptide substrate (BMP Smad C-terminal tails) due to allosterically altered conformations of type I active sites within heterohexameric cytoplasmic signaling complexes—assembled noncanonically by activin-type II receptors extracellularly—is hypothesized to produce a gain of function of the R206H mutant protein responsible for episodic heterotopic ossification in FOP.

## 1. Introduction

The purpose of this study, commenced in 2006 with the identification of the recurrent R206H mutation causative of FOP [1], is to understand the protein structural–functional basis for the gain of function by the ACVR1/ALK2 receptor kinase eliciting sporadic heterotopic ossification. Toward that end, in 2007, a reliable structure-based (TβR-I/ALK5) [2] homology model was generated that showed the mutation was located adjacent to two binding partners, the inhibitory protein FKBP12 and the substrates of ALK kinases, the Smad proteins [3]. Diminished or lost binding by the inhibitory protein, increased affinity for or interaction with the immediate downstream effector, or both appeared to be the simplest bases for the gain of function by the recurrent mutant.

Unfortunately, Smad protein substrate could not be analyzed, because phosphorylation in vitro cannot be detected. Assays of ALK protein kinase activities require surrogate substrates, dephosphorylated casein polypeptides, in descriptions of commercial sources and as reported by academic laboratories. That said, the pioneering work by Huse et al. [4] employed semi-synthetic, tetra-phosphorylated TβR-I/ALK5 to analyze kinase activity with respect to Smad protein substrate, leading to a longstanding paradigm, the inhibitor-to-substrate-binding switch for the role of the metastable helix–loop–helix (HLH) element (also referred to as the GS domain from protein sequence alignments) preceding the highly conserved protein kinase domain.

A main conclusion of the investigations culminating in the inhibitor-to-substrate-binding switch hypothesis was best summed up by the final statement of the introduction of the paper: “Our studies indicate that phosphorylation activates TβR-I not by increasing the overall activity of the kinase domain, but rather by converting the GS region into an efficient recruitment motif for the proper substrate”. Additionally, in a later *Cell* review article entitled “Conformational Plasticity of Protein Kinases” [5], Huse and Kuriyan concluded that “When unphosphorylated, the GS region inhibits the kinase activity of TβR-I, but only when bound by the inhibitory protein FKBP12”. Hence, the HLH regulatory element (GS domain) was regarded as serving solely passive roles as the site of binding of the two protein partners, substrate and inhibitor.

In keeping with the latter, Song et al. [6] concluded in 2010 that impaired binding (approximately 2-fold) by FKBP12 to R206H ACVR1 in murine myogenic C2C12 cells in part (in addition to altered protein expression and distribution) might have contributed to mild activation of osteogenic BMP-signaling in extraskeletal sites such as muscle, leading to delayed and progressive ectopic bone formation. Similarly, Chaikuad et al. [7] highlighted in 2012 that “Disease mutations break critical interactions that stabilize the inactive ALK2-FKBP12 complex leading to kinase activation”. Thus, prevailing views of the molecular basis for R206H gain of function remained in line with the initial paradigm, the inhibitor-to-substrate-binding switch.

Contemporary with those of Song et al. [6] and Chaikuad et al. [7], a rigorous study was conducted that showed FKBP12 binding was not severely reduced but was rather diminished only several-fold [8]. Briefly, in vitro interaction analyses were performed with highly purified recombinant wild-type and R206H kinases and FKBP12 protein. Qualitative and quantitative analyses using native polyacrylamide gel electrophoresis, HPLC size-exclusion chromatography and an optical biosensor (BLI, Bio-Layer Interferometry) provided orthogonal means of assessing relative and absolute binding affinities, which differed by approximately 3-fold. Since the interaction of FKBP12 with ALK receptors was hypothesized by Donahoe and coworker [9] to act as a gradient reader in morphological contexts, the possibility remained open that the modest perturbation of inhibitory protein binding also had a modest effect on heterotopic ossification. However, despite convincingly clear results, cause and effect could not be ascertained and remained speculative.

Subsequently, with an *ACVR1^R206H^* mouse, Hatsell et al. [10] showed that ALK2 R206H gained responsiveness to the noncanonical ligand activin A, which was both necessary and sufficient for driving heterotopic ossification in the FOP mouse model of the genetic disorder. Moreover, they observed that wild-type ACVR1 was not converted into an activin-responsive receptor by abrogating FKBP12 binding with the competitive inhibitor FK506, whereas signaling from canonical ligands was indeed enhanced. So, while FKBP12 diminished signaling by the wild type, diminished binding to the mutant kinase was thus convincingly shown to be insufficient to account for the gain of function leading to FOP pathology.

One should note that the switch hypothesis was arrived at with the investigation of TβR-I/ALK5; however, ALKs behave disparately, as evidenced by different modes of ligand binding and mechanisms of cooperative assembly of signaling complexes. That is, TGFβ ligand-mediated signaling complexes cooperatively assemble via direct interactions between receptor extracellular domains [11], whereas BMP ligand-mediated counterparts rely on restriction to two dimensions in the plasma membrane, as shown by kinase-domain-truncated but membrane-anchored ALK2 constructs cooperatively assembling on BMP-7 with ACVR2A [12]. Perhaps most importantly though, mechanistic conclusions of the studies might only be relevant for wild-type ALK kinases, not the gain-of-function R206H mutant and caALK designed variants, a crucial distinction.

A seminal finding that has advanced the understanding of the molecular mechanisms underlying the gain of function by the R206H mutant and caALK2 is the observation by Knaus and coworkers that heterodimeric complexes of types I and II BMP receptor kinases pre-form in the cytoplasm in the absence of ligand recruitment extracellularly [13,14,15,16]. Note that TβR-I/ALK5 and TβR-II, the singular counterparts of TGFβ signaling, do not appear to pre-form in a comparable manner, another aspect setting apart BMP ALKs from TβR-I/ALK5. Pre-formed receptor kinase heterodimers appear to be a conserved feature of BMP signaling, in that Le and Wharton showed a requirement for a type II receptor with the hyperactive human ACVR1/ALK2 R206H mutant in a *Drosophila* model of FOP [17]. Although the mildly or constitutively active type I ALK2 kinases require an upstream type II receptor kinase, Bagarova et al. [18] reported that the protein scaffold, not phosphorylation activity, is both necessary and sufficient for ligand-independent gain of function by the recurrent mutant and caALK2 variant. In addition to the genetic and cellular-based evidence, complex formation was shown between recombinant ALK2 and BMPRII kinases by analytical ultracentrifugation and by size-exclusion chromatography. More recently, Natalia Jura and coworkers employed hydrogen–deuterium exchange mass spectrometry (HDX-MS), small angle X-ray scattering (SAXS) and molecular dynamics (MD) simulations to structurally characterize complex formation between BMPRII and ALK2 [19].

We have also observed heterodimeric interaction by the two kinases in vitro by native PAGE in our characterization of allosteric destabilizers of ALK2 [20]. Furthermore, a computationally docked BMPRII–ALK2 heterodimer model was generated that appeared sound structurally [21], and functionally, in that the predicted three-dimensional complex was consistent with BMPRII-imparted resistance of ALK2 to destabilization by allosteric compounds that docked and apparently bound adjacent to the protein–protein interface [20].

The specific target of destabilizing compounds was a key allosteric hub for transmitting conformational changes regulating protein kinase activities, the loop connecting the C-terminus of the αC helix to the N-terminus of the β4 strand [22]. Though protein kinases can be described as two lobes (N and C) that are hinged at the ATP-binding cleft and rotate globally along the long axis relative to one another, a more nuanced understanding of the mechanisms of activation has arisen from the ground-breaking work of Susan Taylor and coworkers, who have shown that allostery and dynamics transmitted along hydrophobic spines sharing the long axis dictate the equilibria between active and inactive states [23,24,25,26,27,28]. Along those lines, via a range of analytical and computational techniques, various gatekeeper mutations in fibroblast growth factor receptors were recently shown to affect the inactive, autoinhibited state of the tyrosine kinase, destabilizing it by weakening the hydrophobic spine, allowing the kinase to dynamically shift to more active forms [29].

In lieu of the inhibitor-to-substrate-binding switch paradigm, polypeptide substrate accessibility was previously put forth to act as the basis for the gain of function by the R206H mutation in FOP. First presented at the 11th International BMP Conference, Harvard Medical School Oct 2016, and later at the FOP Drug Development Forum, Sardinia, Italy Oct 2017, the hypothesis has also been discussed in two publications [20,21], and most recently in a third in the context of the variant form of FOP due to an even more rare R375P ACVR1/ALK2 mutation [30]. However, up until now, the hypothesis has not been published with the supporting data. In this Special Issue contribution, in vitro results are reported showing that the metastable HLH regulatory element actively suppresses the phosphotransferase activity of the enzyme, evidently because perturbation elevates kinase activity at the distant active site. Furthermore, a putative three-dimensional model of the heterohexameric signaling complex [21] is depicted herein to support the hypothesis that accessibility to polypeptide substrate (BMP Smad C-terminal tails) due to allosterically altered conformations of type I active sites produces the relatively mild gain of function of the R206H mutant protein responsible for episodic heterotopic ossification in FOP.

## 2. Materials and Methods

### 2.1. Materials

Smad-surrogate substrates (caseins) for kinase activity assays by autoradiography were purchased from SignalChem (Richmond, BC, Canada) exclusively. Solubilized mixtures of partially dephosphorylated, intrinsically disordered casein polypeptides were unstable upon prolonged storage; thus, they were divided into small aliquots and frozen for moderate durations for subsequent use.

### 2.2. Expression and Purification of Recombinant Proteins

#### 2.2.1. ACVR1/ALK2 Receptor Kinases

Cytoplasmic domains of wild-type, R206H mutant, Q207D variant and 208 Ntrunc ACVR1/ALK2 receptor kinases were expressed from synthetic cDNAs (GenScript, Tokyo, Japan) in Sf21 insect cells after infection with high-titer recombinant baculovirus stocks produced with the Invitrogen Bac-to-Bac (DH10Bac bacmid and pFastBacHT vectors). The kinase forms, fused N-terminally with a 6xHis affinity tag and a tobacco etch virus (TEV) protease cleavage site, were isolated from sonicated and cleared insect cell lysates by Ni^2+^-NTA (Qiagen, Hilden, Germany) affinity chromatography. After binding in batch, the recombinant proteins were eluted, cleaved with TurboTEV protease (Eton Bioscience, San Diego, CA, USA), and purified to near-homogeneity by a subsequent reverse affinity step. The forms spanned from Thr172 to the native C-terminus, Cys509, with the exception of the truncated form, which spanned from Ile208 to Cys509.

#### 2.2.2. FKBP12 Proteins

Recombinant 6xHis-TEV-FKBP12 (GenScript) was expressed with the pET system (BL21DE3 and modified pET15b), isolated from sonicated and cleared *E. coli* lysates by affinity chromatography, cleaved with TEV and purified by reverse affinity. A charge variant (substitutions yielding six net negative charges) with a negative pI was engineered through modification of the flexible N-terminal affinity tag (6xHis-TEV) that did not affect ALK2 binding but allowed for analyses by native PAGE. The charge variant for native gel assays was purified by a single affinity step and not subjected to proteolysis.

#### 2.2.3. BMPRII Kinase

BMPRII kinase domain was expressed in *E. coli* as a small ubiquitin-like modifier (SUMO) fusion protein (8xHis-SUMO-TEV; GenScript) from a synthetic cDNA and purified from a sonicated and cleared lysate (containing a soluble fraction of recombinant protein) by forward and reverse nickel affinity chromatography. The nearly homogeneous, monomeric kinase was purified by a subsequent polishing step by S-75 size exclusion chromatography (Superdex, Pharmacia/GE).

#### 2.2.4. Golgi Casein Kinase

Recombinant Golgi Casein Kinase (mouse FAM20C protein [31]) was expressed in insect cells with the Bac-to-Bac baculovirus expression system (Invitrogen, Waltham, MA, USA). The N-terminus of secreted mouse FAM20C was fused with a baculovirus signal sequence (from gp67 envelope glycoprotein), 6xHis affinity tag, SUMOstar solubilizing fusion partner and a TEV cleavage site. The engineered fusion gene was inserted into the pFastDual vector (Invitrogen) downstream of a polyhedrin promoter. In addition, a GFP cDNA was inserted downstream of a P10 promoter to determine the extent of baculoviral infection in insect cells. DH10Bac *E. coli* cells (Invitrogen) were transformed with the dual construct, which was subsequently introduced into BacMid via homologous recombination.

GCK/FAM20C BacMid was extracted from DH10Bac *E. coli* cells and transfected into Sf21 insect cells (Invitrogen) to produce recombinant baculovirus. Recombinant GCK/FAM20C was secreted into the SFX-insect cell culture medium (Hyclone). After two rounds of scale-up to increase the titer, the cell culture medium was collected and GCK/FAM20C was enriched by Ni-NTA chromatography. Following cleavage with TurboTEV, reverse Ni-NTA purification was performed to remove TEV protease and the N-terminal fusion tag (6xHis-SUMOstar-TEV site) and yield nearly homogeneous GCK/FAM20C. The mammalian Golgi Casein Kinase reproducibly yielded large, highly birefringent crystals with sharp edges, indicative of the purity and stability of the preparation (albeit poorly diffracting due to apparent internal disorder).

### 2.3. In Vitro Kinase Assay, SDS-Polyacrylamide Gel Electrophoresis and Autoradiography

Denaturing PAGE was performed with a standard Tris-glycine (pH 8.3) system per Laemmli. ALK2 kinase was radiolabeled with [γ-33P]ATP (NEN-PerkinElmer, EasyTide, NEG602K250UC) diluted into cold ATP (100 μM) and analyzed by Coomassie staining and by autoradiography of SDS-polyacrylamide gels air-dried between sheets of cellophane. Proteins were separated through 4% stacking/12% resolving acrylamide gels. Further specific method details are not available.

### 2.4. Native Polyacrylamide Gel Electrophoresis of Proteins

Nondenaturing PAGE of native proteins for analysis of complex formation or autophosphorylation was performed as previously described [11]. Briefly, the same system as for denaturing PAGE was used, but SDS was omitted from the sample, running and loading buffers (Invitrogen-Novex recipes). As for denaturing PAGE, proteins were electrophoresed through 4% stacking/12% resolving acrylamide gels. For autophosphorylation assays, kinases were incubated with 1 mM ATP and 10 mM divalent cation for varying times at 30 °C. Reactions were terminated with EDTA prior to analysis by native PAGE.

### 2.5. Non-Radioactive Kinase Activity Assay by ADP-Glo Method

Rather than assessing radioactive ATP γ-phosphoryl group transfer, the ADP-Glo Kinase Assay (Promega, Madison, WI, USA) indirectly quantitated ADP produced by subsequent conversion into ATP, which is used to generate light in a luciferase reaction followed by white-light luminescence (RLU). Assays were performed per the instructions of the manufacturer. To ensure accuracy and reproducibility (reduction in scatter and noise), food colorings were added to components prior to mixing, which as a result was readily visualized as thorough and complete by elimination of input colors (red, green) and generation of a new one (yellow). Further specific method details are not available.

### 2.6. Western Blot Analyses of BMP Signaling in Murine Primary Culture Cells

MEFs were isolated from 13.5 dpc mouse embryos and used as described [32]. Cells were cultured in Dulbecco’s modified Eagle’s medium (DMEM, Gibco, Grand Island, NY, USA) containing 10% fetal calf serum (Invitrogen). At least three individual embryo samples were used for experimental replicates.

For signaling assays, MEFs were cultured in DMEM without serum for 2 h prior to adding BMP4 (100 ng/mL; R&D Systems, Minneapolis, MN, USA) for positive control samples or FK506 (Sigma, St. Louis, MO, USA) at 0.001, 0.01, 0.1 and 1.0 µM for 1 h.

For immunoblot detection, total cell protein was recovered using M-PER containing Halt Protease and Halt Phosphatase Inhibitor Cocktails and quantified using a Pierce BCA Protein Assay Kit (Thermo Fisher Scientific, Waltham, MA, USA). Proteins were electrophoresed through 10% SDS-polyacrylamide gels and transferred to nitrocellulose (Invitrogen). Membranes were blocked in 5% milk and incubated with primary antibodies against phosphorylated Smad1/5/8 (1:750) and β-actin (1:3000) (Cell Signaling Technology, Waltham, MA, USA), at 4 °C overnight. Bound antibodies were detected with anti-rabbit horseradish peroxidase-conjugated secondary antibody (1:6000) (Cell Signaling Technology) at room temperature, 1 h. Detected proteins were imaged with Immobilon Chemiluminescent HRP Substrate (Millipore, Billerica, MA, USA) and quantified using ImageJ Software.

### 2.7. Western Blot Analyses of BMP Signaling by ALK2 Forms in Drosophila S2 Cells

The ALK^ΔGS^ construct was generated using Quikchange Site-Directed Mutagenesis to remove the GS domain (T189–Q207) from wild-type ALK2. Following Sanger sequencing to confirm the deletion, the ALK2^ΔGS^ was shuttled using LR reaction (Invitrogen) from the pDONR221 vector to the pAWF vector, which carries a C-terminal 3xFLAG tag and an actin 5C promoter for constitutive expression in cell culture. Drosophila S2 cells were cultured under standard conditions (M3 media pH 6.5, supplemented with 10% IMS, 2% FBS). For each transfection, 8 × 10^6^ cells (at 4.0 × 10^6^/mL) were used. Cells were cotransfected with 700 ng pAC FLAG-Mad (a gift of M. O’Connor, Minneapolis, MN, USA) and 300 ng of one of the following constructs: pAWF ALK2^ΔGS^, pAWF ALK2 or pAWF ALK2^R206H^ [17]. Seven days post-transfection, transfected cells were harvested by centrifuge (5 min spin at 0.4 rcf). The supernatant was discarded and cell pellets were lysed with 0.5 volumes of 2× SDS buffer. Cell lysates were run on a 12% SDS-PAGE gel, and protein was transferred onto a PVDF membrane by semi-dry transfer. The following antibodies were used for Western blot analysis: anti-PS3 Rb (1:1000, Epitomics, Burlingame, CA, USA) to detect pMad [17] and anti-FLAG M2 (1:1000, Sigma) to detect total receptor and Mad expression.

### 2.8. Determination of K_m_ for ATP

K_m_ values for ATP of autophosphorylation of ALK2 kinase forms were determined using the luminescence-based ADP-Glo assay described above. To calculate values, the data were fitted either through linear or non-linear regressions for comparison. Linear regressions were to double-reciprocal plots of data in Excel and values calculated from equations. Non-linear regressions were fitted directly to curves and values calculated automatically by built-in Michaelis–Menten analysis with GraphPad Prism.

## 3. Results

### 3.1. Diminished FKBP12 Interaction Does Not Appear Causative of Kinase Dysregulation

#### 3.1.1. Native PAGE Analysis of FKBP12 Binding to ALK2 Kinase Forms

**Figure 1:**
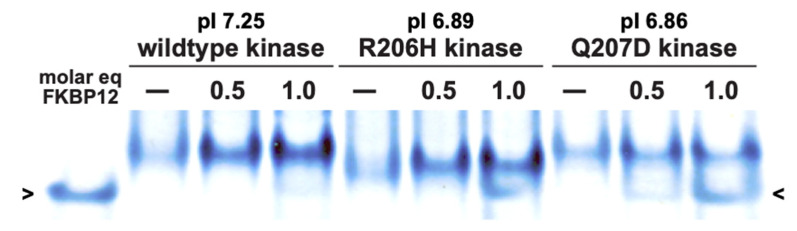
Native PAGE analysis of FKBP12 interaction with ALK2 kinase forms. Left to right: wild type, FOP mutant, constitutively active substitution variant. Values of 0.5 or 1.0 refer to molar equivalents of FKBP12. Open arrowheads mark position of free FKBP12 migration. Note disparate levels of unbound inhibitory protein not taken up into complexes.

As introduced above, a thorough study of FKBP12 binding to wild-type and R206H mutant kinases was conducted [8]; however, it did not include the constitutively active engineered variant, Q207D. Though not examined by the multiple orthogonal methods here, inhibitor binding to recombinant Q207D kinase was compared to wild-type and recurrent mutant proteins by native polyacrylamide gel electrophoresis (Figure 1). The affinity of FKBP12 for Q207D kinase was diminished relative to the wild type, comparable to the affinity for R206H, as evidenced by residual unbound FKBP12 protein at molar equivalence in both cases. A trace of free inhibitor was even seen at half-molar equivalence with Q207D that was not with R206H, indicative of slightly lower affinity. The migration rate of the variant was slower than anticipated by the calculated isoelectric point (6.86). The mutant, which lost a positive charge relative to the wild type, correspondingly migrated at a faster rate, which should have been mirrored by the variant. Nonetheless, the conclusion drawn from the native PAGE results remained clear, i.e., that the robustly constitutively active variant was bound by the inhibitory protein with an affinity similar to that of the mildly activated mutant, and hence the disparately higher levels of activity relative to the wild type were unlikely due to altered FKBP12 binding.

#### 3.1.2. In Vitro Kinase Inhibition by FKBP12 and Comparison with Dorsomorphin

**Figure 2:**
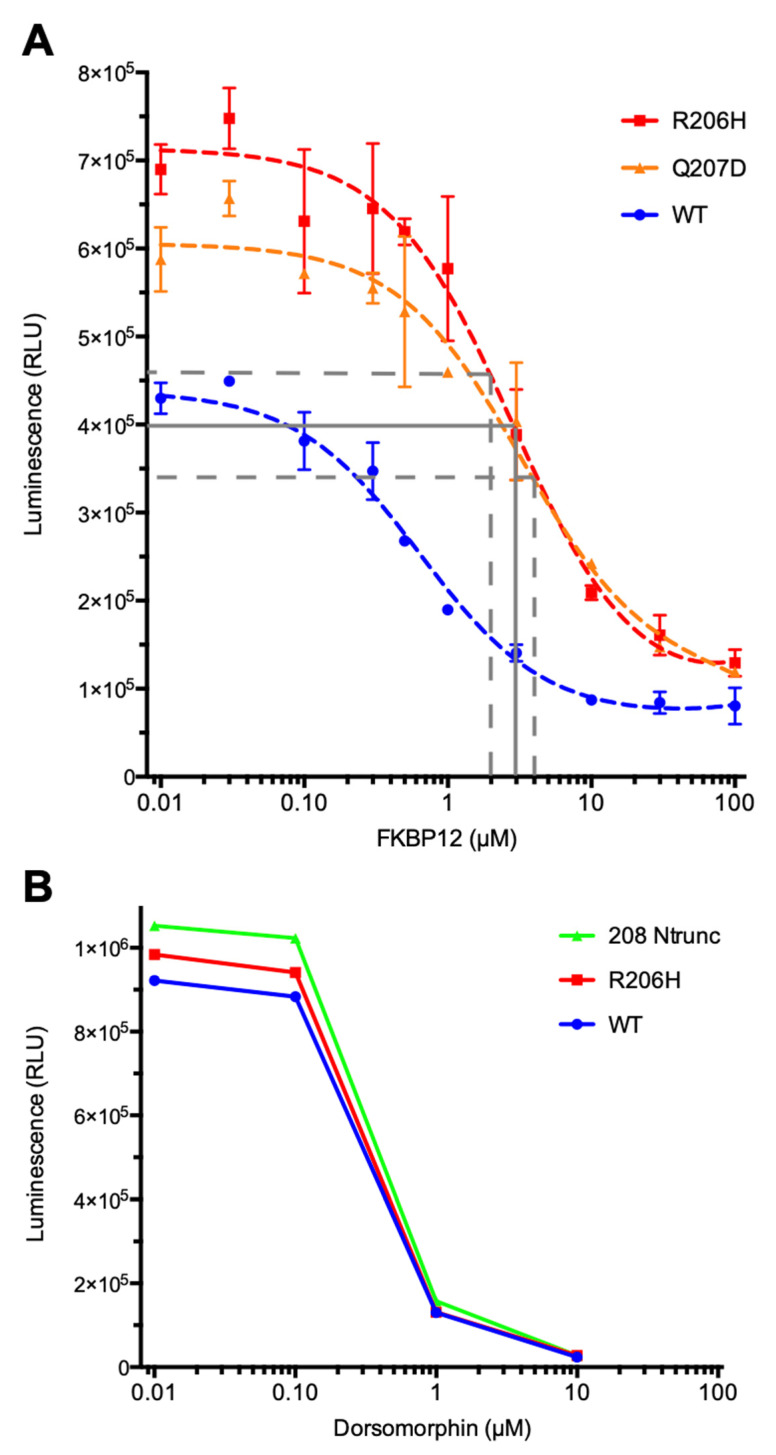
In vitro kinase inhibition by FKBP12 protein and ATP-competitor dorsomorphin of kinase forms. (**A**) At cellular concentration range of FKBP12 (solid grey line), mutant and variant activities (dashed grey lines) were comparable to unbound wild type. (**B**) The ATP-competitive inhibitor dorsomorphin can fully inhibit. 208Ntrunc is a variant form with deletion of the so-called GS region preceding the protein kinase domain.

In addition to the qualitative assessment of FKBP12 affinities for the ALK2 protein forms, a quantitative analysis was conducted by measuring inhibition of the kinase activities of the three (Figure 2). The non-radioactive ADP-Glo (Promega) method that uses luminescence (RLU) rather than radioactive ATP γ-phosphoryl group transfer to quantitate ADP produced was employed. Trans-autophosphorylation activities of kinases (Section 3.5, below) were measured, since as noted above, Smad MH2 phosphorylation in vitro was undetectable. In the absence of an FKBP12 inhibitor, the mutant and variant kinases were more active than the wild type, approximately 1.7- and 1.4-fold, respectively (Figure 2A). Interestingly, the more weakly activated mutant exhibited more autophosphorylation activity than the robustly activated caALK2 variant. As the concentration of FKBP12 was increased to that of an estimated cellular level (3 μM), the activities of both the mutant and variant were inhibited to approximately the level of the uninhibited, FKBP12-free wild-type kinase. With saturating amounts of FKBP12 (100 μM), none of the three were completely inhibited. In contrast, the ATP-competitive inhibitor dorsomorphin quenched ALK2 kinase activities completely at saturation (10 μM) (Figure 2B).

#### 3.1.3. Effects of FK506 Competition for FKBP12 Binding on BMP Signaling in Primary Cells

**Figure 3:**
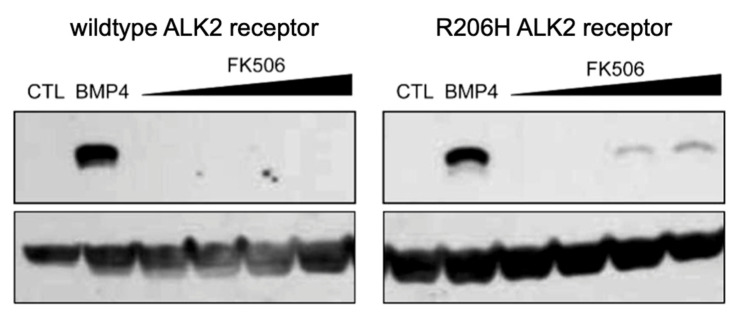
Wild-type and R206H ALK2 signaling in response to FK506, inhibitor of FKBP12. FK506 competes with the type I BMP receptor HLH/GS domain for FKBP12 binding, thereby dissociating FKBP12 from the receptor and promoting Smad phosphorylation. Immunoblot detection of pSmad1/5/8 induction following treatment of mouse embryonic fibroblasts with increasing concentrations of FK506 (0.001, 0.01, 0.1, 1.0 µM) is shown minus BMP4 except for positive controls (BMP4; 100 ng/mL). pSmad1/5/8 was normalized to β-actin (lower panels).

To assess the effects of diminished binding of FKBP12 to the mutant receptor kinase with respect to BMP signaling, Smad phosphorylation was analyzed in primary culture cells (Figure 3). Embryonic fibroblasts from wild-type or R206H mutant mice were subjected to increasing amounts of FK506, a competitive inhibitor of FKBP12 binding, along with negative (no ligand, CTL) and positive (+BMP4 ligand) controls. Strikingly, even at the highest level of the competitor, the wild-type receptor kinase did not phosphorylate Smad substrates above background levels. With respect to the R206H mutant, weak phosphorylation activities were observed at the two highest concentrations, although Smad phosphorylation was undetectable at lower levels and in the absence of the competitor. These findings were consistent with the results of autophosphorylation assays (Figure 2A) in that physiological levels of FKBP12 inhibited the mutant to the level of the unbound wild type and activities rose at lower levels of bound FKBP12 (at higher FK506 concentrations).

In line with our findings, Song et al. [6] showed that FK506 treatment alone was not sufficient to produce positive staining for alkaline phosphatase (a common marker of BMP-induced osteoblast differentiation) in wild-type ALK2-transfected cells, whereas R206H-overexpressed cells were alkaline phosphatase-positive. However, although 0.5 μM FK506 was sufficient to increase downstream transcriptional (*Alp*) activity in Q207D-transfected cells, the treatment was insufficient to stimulate such activity over a threshold level in the mildly activated R206H-transfected cells.

Taken together, these results indicated that several-fold diminished binding of FKBP12 to the mutant does not lead to leaky signaling in transient transfection experiments [6] or in a physiologically relevant context, i.e., primary cells from transgenic *ACVR1^R206H^* mice. Above all, modestly diminished FKBP12 binding was ruled out as the underlying basis of FOP, in keeping with the in vivo findings of Hatsell et al. that abrogation of FKBP12 binding was insufficient to promote heterotopic ossification in an FOP mouse model [10].

### 3.2. BMPRII Kinase Interaction Is Unaltered and Hence Not Causative of Dysregulated Signaling

#### Native PAGE Analyses

As introduced above, a seminal finding by Knaus and coworkers has provided insight into molecular mechanisms underlying the gain of function by the R206H mutant and caALK2, i.e., the observation that heterodimeric complexes of types I and II BMP receptor kinases pre-form in the cytoplasm in the absence of ligand recruitment extracellularly. As also mentioned, we have previously observed heterodimeric interaction between recombinant ALK2 and BMPRII kinases in vitro by native polyacrylamide gel electrophoresis in the course of our characterization of allosteric destabilizers of ALK2 [20].

**Figure 4:**
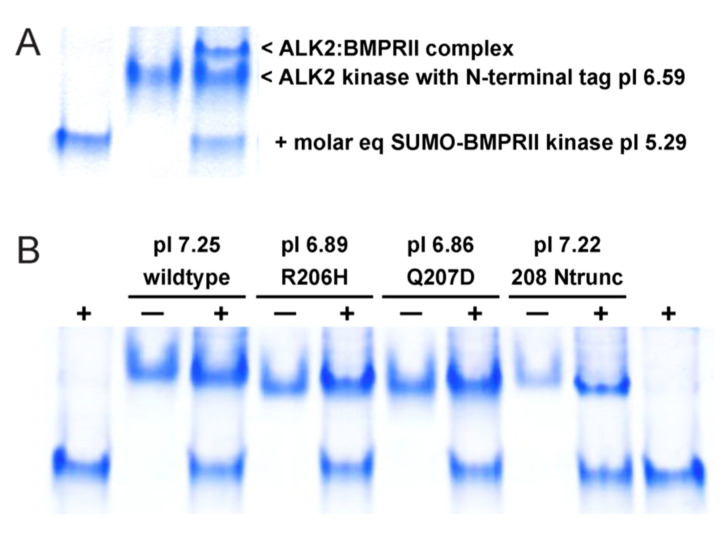
Native PAGE analysis of BMPRII interaction with ALK2 kinase forms. (**A**) Complex formation with SUMO-BMPRII kinase fusion protein (pI 5.29, MW 49.8 kDa) and (**B**) with BMPRII kinase (pI 5.25, MW 36.2 kDa). Plus and minus signs refer to BMPRII in each lane.

To determine whether ALK2 kinase forms interacted differentially with BMPRII kinase, we employed native PAGE to analyze the propensities to form complexes (Figure 4). As seen before, mixing the two recombinant kinases produced a third species, the complex, which migrated more slowly than the free components (Figure 4A). Note that the complex did not form to completion, indicative of weak protein–protein interaction at the heterodimer binding interface. However, in the case of full-length receptor kinases anchored in the two-dimensional plasma membrane, the complexes would likely form to completion, in keeping with the membrane-mediated mechanism of cooperative assembly hypothesized for ALK2-containing and other BMP signaling complexes [12].

To ensure that the N-terminal tag (small ubiquitin modifier (SUMO)) employed for overexpression and solubilization did not interact with BMPRII kinase, the fusion partner was cleaved and removed from preparations of the four ALK2 forms. As a result, though, free ALK2 forms and respective complexes with BMPRII nearly comigrated (Figure 4B). Hence, complex formation required assessment of slight alterations in migration rates, intensities and consumption of free BMPRII kinase protein. Overall, little if any difference could be discerned with respect to the tendency to heterodimerize in vitro, effectively eliminating alterations in interaction with type II kinase—such as enhanced complex formation—as a basis for the gain of function by the R206H mutant or Q207D caALK2 variant. That said, the formal possibility remained open that ACVR2A/ActRIIa kinase might behave differently, though the likelihood is low, since structure-based homology models of both heterodimers were highly similar throughout and most importantly at the putative dimer interface.

With respect to the homology models of heterodimers, the native PAGE result with the truncated form provides support of reliability. Specifically, the removal of the HLH segment did not interfere with complex formation, consistent with our three-dimensional, computationally docked model, which does not include the segment at the predicted protein–protein interface.

### 3.3. Elevated In Vitro Kinase Activity of R206H Mutant Suggestive of Basis for Gain of Function

#### 3.3.1. In Vitro Kinase Activities Assayed by Autophosphorylation and with Surrogate Substrate

**Figure 5:**
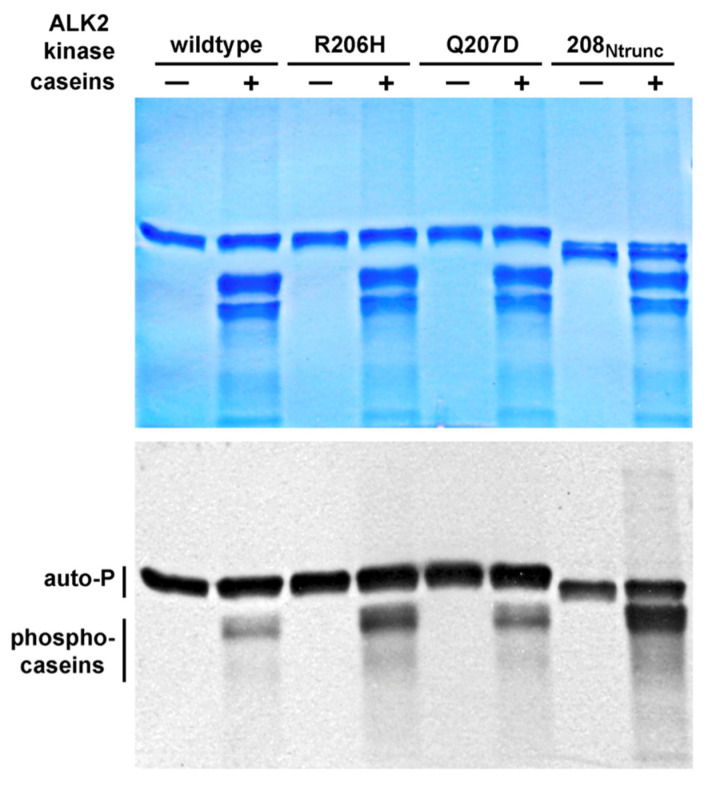
In vitro kinase activities assessed by autophosphorylation and with casein substrate. Coomassie-stained SDS-PAGE gel (**upper** panel) and [γ-^33^P]ATP autoradiogram (**lower** panel) of equimolar ALK2 kinase forms at near-homogeneity. Partially dephosphorylated casein substrate is a mix of four polypeptide forms.

As presented above (Section 3.1.2), mutant and caALK2 variant kinases were more active than the wild type with respect to transphosphorylation (Figure 2A), suggesting a basis for the gain of function of both, though the mildly activated mutant exhibited more autophosphorylation activity than the robustly activated caALK2 variant. To further investigate and compare the kinase activities of the two forms as well as the truncated variant, autoradiography was employed with [γ-^33^P]ATP and casein polypeptide substrates, in addition to autophosphorylation (Figure 5).

Casein, a major component of milk, is made up of four different polypeptides, α_S1_-, α_S2_-, β- and k-casein, that are proline-rich yet have no well-defined secondary and tertiary structures and possess clusters of phospho-serine and phospho-threonine residues. Partially dephosphorylated (enzymatically) casein is perhaps the most commonly employed substrate for assaying protein kinases in vitro. In actuality, caseins serve well as surrogate Smad substrates since the proline-containing C-terminal tails of Smads, the site of activating phosphorylation by ALK kinases, is loosely structured and composed of motifs of neighboring serine and or threonine residues.

In keeping with the relative transphosphorylation activities quantitated previously by the ADP-Glo assays (Figure 2A), R206H kinase phosphorylated the surrogate Smad substrate pronouncedly more than the wild type, with Q207D/caALK2 being intermediate in activity. Strikingly, however, with truncation of the regulatory HLH element (208 Ntrunc), caseins were phosphorylated at perhaps twice the level as by the FOP mutant kinase, R206H. Hence, perturbation by point mutation and by deletion both clearly allowed for an increase in basal kinase activities with respect to the wild type, meaning that the HLH segment did not act statically as an inhibitory protein binding partner, but actively as a negative regulator of activity.

In conclusion, with respect to heterotopic ossification caused by ALK2 R206H, elevated basal activity appears responsible for the relatively mild gain of function of the mutant kinase, but not the robustly activated, aspartate-substituted caALK2 form, which must achieve a high level of activity due to a different mechanism of action.

#### 3.3.2. In Vitro Kinase Activities Assayed with Surrogate Substrate Compared to the Dedicated Cellular Enzyme, Golgi Casein Kinase

Because of the heterogeneous nature of the casein polypeptide substrates, we hypothesized that increased levels of phosphorylation by the HLH-perturbed forms were due to an increase in the number of sites modified. That is, the increase was not with respect to rate, but accessibility to more sites or clusters of dephosphorylated serine or threonine residues along the unstructured polypeptides. Toward that end, we assayed increasing amounts of wild-type and R206H mutant kinases to confirm that the reactions had reached completion. We also determined whether the caseins were heterogeneous not only structurally but functionally, that is to say, whether the compositions contained numerous sites of varied accessibility. The latter question was addressed by utilization of the dedicated cellular enzyme, Golgi Casein Kinase, which phosphorylates secreted phosphoproteins such as those found in milk [33].

**Figure 6:**
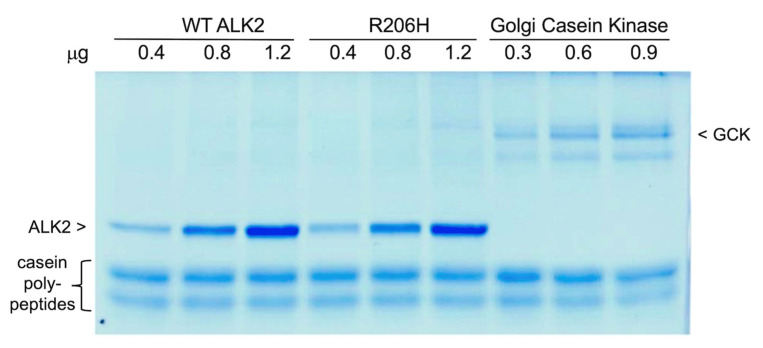

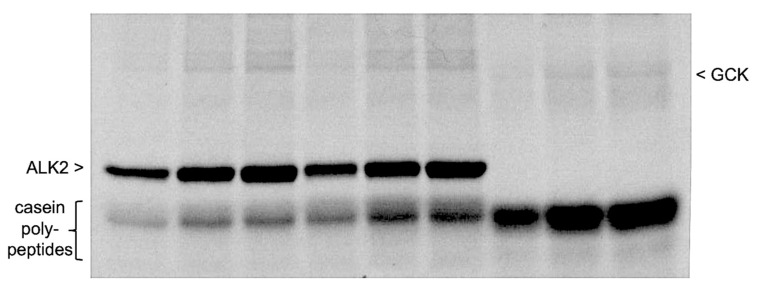
In vitro kinase activities assessed with casein substrate, comparing wild-type and mutant ALK2 forms to the dedicated cellular enzyme, Golgi Casein Kinase. Coomassie-stained SDS-PAGE gel (**upper** panel) and [γ-^33^P]ATP autoradiogram (**lower** panel).

With respect to the former question, as can be seen in Figure 6, the reactions reached completion at the two higher amounts of kinases. As before, the FOP mutant kinase was clearly more active than the wild type with casein substrate. With respect to the latter question, relative to Golgi Casein Kinase, the R206H mutant modified a fraction of the apparent sites of the heterogeneous substrate, and wild-type ALK2 kinase modified a fraction even smaller still. This finding strongly suggests that accessibility to surrogate polypeptide substrate is mildly increased by perturbation of the HLH regulatory element by the R206H point mutation, which in vivo is hypothesized to allow for increased accessibility to BMP Smad C-terminal tails within cytoplasmic signaling complexes assembled noncanonically by activin-type II receptors extracellularly.

### 3.4. Analysis of BMP Signaling by Wild-type, Mutant and Regulatory Subdomain-Deleted ALK2 Receptors in Drosophila S2 Cells

**Figure 7:**
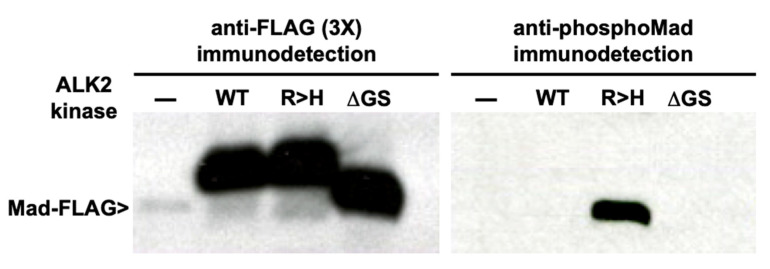
Western blot analyses of BMP signaling by ALK2 forms in Drosophila S2 cells. Comparable, high-level expression of ALK2 constructs (**left** panel). Robust signaling observed with less active R206H, but undetectable with significantly more active truncated (ΔGS or HLH-deleted) form of the receptor kinase (**right** panel).

Because truncation of the negatively acting HLH regulatory element dramatically increased the level of surrogate substrate phosphorylation (Figure 5), the question arose whether deletion of the segment from within a receptor kinase in the cell would be sufficient for comparably high levels of signaling. To address the possibility, a cell-based analysis with *Drosophila* S2 cells was performed, comparing the levels of *Drosophila* Smad (Mad) phosphorylation after transient transfection with wild-type, R206H mutant and HLH-deleted (ΔGS) ALK2 forms. As shown in Figure 7, the ALK2 constructs were expressed at comparable and at high levels. As anticipated, no phosphorylation of Mad substrate was detectable by the wild type in the absence of stimulation by a ligand. Also as anticipated, as seen before, the human mutant kinase phosphorylated Mad in the S2 cells; however, the more extreme perturbation—deletion of the negative regulatory element—failed to produce detectable Mad phosphorylation.

In conclusion, basal kinase activities alone were not correlated with gain of function. Deletion of the second helix of the HLH element is predicted to eliminate Smad interaction; thus, the truncated form, though highly active in vitro with casein substrate, does not signal detectably. Similarly, caALK2 was less active than the mutant and marginally more so than the wild type with casein, although it signals robustly and constitutively in vivo. In other words, protein–protein interactions in vivo within the assembled heterohexameric (two type I receptors, two type II receptors, two Smad substrates) signaling complex appear necessary, while increased phosphotransferase activity alone, i.e., independent of complex formation, was insufficient.

### 3.5. Native PAGE Analyses of Autophosphorylation Extents and Divalent Cation Preferences

Native polyacrylamide gel electrophoresis as an assay for kinase activities by means of trans-autophosphorylation was a convenient method employed early on and throughout our studies. However, the nature of the modification proved to be unexpected. Because the established site of ALK phosphorylation by the upstream constitutively active type II receptor kinases in cellular assays was the cluster of serine and threonine residues in the glycine–serine-rich (GS) loop of the helix–loop–helix regulatory segment, we anticipated that the ALK forms would be multiply transphosphorylated within the same site.

**Figure 8:**
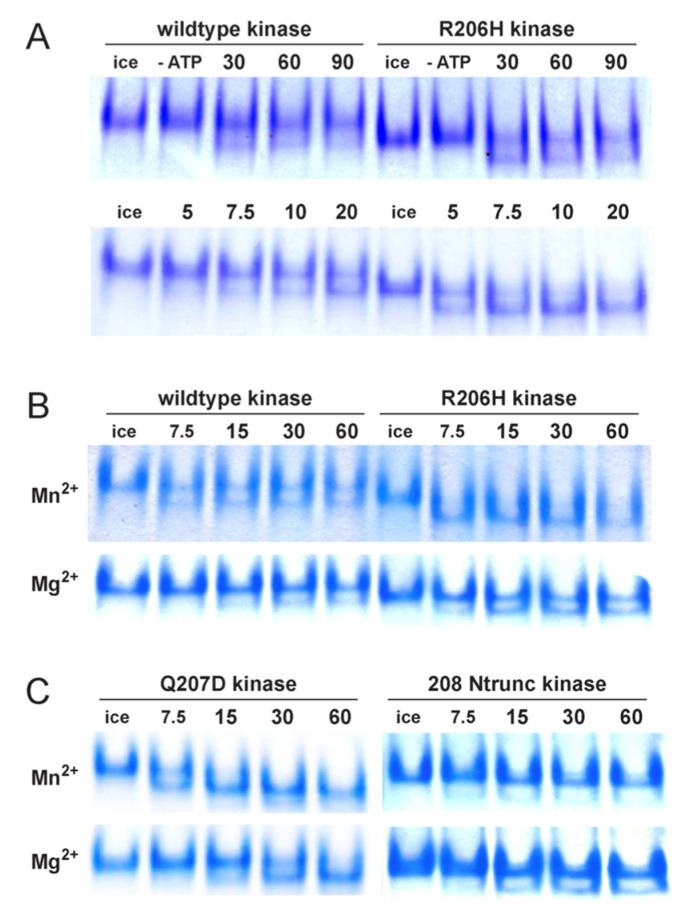
Native PAGE analyses of autophosphorylation rates, extents and divalent cation preferences. (**A**) Reactions with freshly prepared wild-type and mutant kinases terminated at 30, 60 and 90 min intervals (*upper panel*) and with aged preparations terminated at intervals of 5, 7.5, 10 and 20 min (*lower panel*). (**B**) Comparison of effects of ATP in complex with Mn^2+^ or Mg^2+^ on rate and extent of autophosphorylation of aged wild-type and mutant (R206H) kinase preparations. (**C**) Comparison of effects of ATP in complex with Mn^2+^ or Mg^2+^ on rate and extent of autophosphorylation of aged caALK2 (Q207D) and regulatory subdomain-truncated (208 Ntrunc) kinase preparations. Divalent cations were both 10 mM.

To our surprise, only a single residue was phosphorylated, as indicated by precursor–product relationships with respect to time with fixed amounts of kinase (Figure 8). Mass spectrometry identified the residue to be Ser 362 in the A-loop, remote from the GS domain (preliminary data). Crystal structures of ALK2 kinase (e.g., PDB 3H9R) show that the A-loop is loosely structured, with the exception of HLH/GS domain-truncated forms, in which the loop adopts a well-structured conformation.

In the case of wild-type kinase, the more negatively charged, singly phosphorylated product migrated at a rate comparable to that of the unmodified mutant, which had a lower isoelectric point due to the loss of an arginine residue which would be positively charged at the pH (8.3) of electrophoresis (Figure 8A). Similarly, in the case of the R206H mutant kinase, the more negatively charged, singly phosphorylated product migrated at an increased rate by an amount comparable to that of the modified wild type.

Interestingly, neither form was phosphorylated to completion, perhaps only about one-third of the wild type and one-half of the mutant and in each case terminating by 30 min, the earliest time point sampled. Of further interest, we found that the fractions of product to precursor increased in each case reproducibly with increased age of preparations. That is, about one-half of aged (stored at 4 °C, weeks to months) wild-type kinase was phosphorylated and nearly all of the mutant was (Figure 8A, lower panel). Additionally, by examining shorter time intervals, we found that the rate of the mutant was approximately twice as fast as the wild type.

Because manganese was shown by Huse et al. [4] to allow for more sensitive detection in vitro of the weak basal activity of ALK kinase, the standard reaction for autophosphorylation included the same, in lieu of the biologically relevant counterpart, magnesium. Hence, we examined the activities of wild-type and mutant kinases comparatively with the two ATP-binding cofactors (divalent cations) (Figure 8B). In keeping with the findings of Huse et al., autophosphorylation activities with magnesium were greatly reduced compared to those with manganese. We also looked at the preferences of the two variants, Q207D/caALK2 and HLH-truncated (Figure 8C). After 15 min, Q207D kinase autophosphorylated to limit with manganese, whereas the product was barely detectable with magnesium. However, in marked contrast to the wild type, mutant and caALK2 variant, the HLH-truncated kinase, with a structured A-loop, was nearly inert with manganese and weakly active with magnesium.

This latter finding clearly highlighted the allosteric effect of perturbation of the HLH element, even more so than in the case of the R206H point mutation, in that dramatic change at the active site, distant from the regulatory subdomain, was observed. Note also that the effects were not mediated passively by inhibitor binding but instead revealed the role of the helix–loop–helix element in actively regulating (autoinhibiting) the ALK2 kinase.

### 3.6. Determination of K_m_ for ATP of Autophosphorylation of ALK2 Kinase Forms by Indirect Measurement of ADP Production

**Figure 9:**
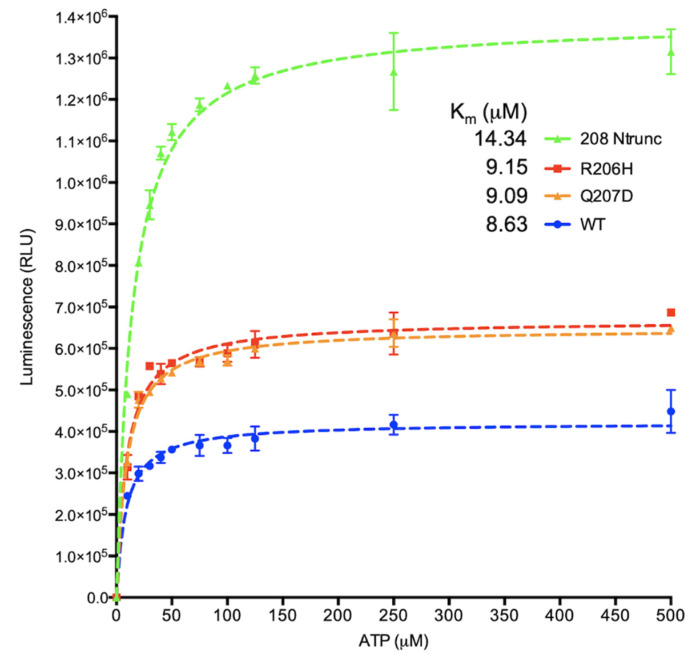
Determination of K_m_ for ATP of autophosphorylation of ALK2 kinase forms by luminescence-based ADP-Glo assay. Data fit by non-linear regression to curves. Given relatively weak γ-phosphoryl transfer to principal site in the A-loop (Ser362), the rate and extent of truncated form are suggestive of hydrolysis of ATP in an open active site rather than phosphotransfer to polypeptide substrate. Divalent cation concentration was 10 mM.

Toward understanding the nature of the changes at the active site of the kinase forms triggered by perturbation of the HLH regulatory element, we compared the affinities for the ATP substrate by determining Km values for the four ALK2 forms (Figure 9). The values for the mutant and Q207D variant were similar, slightly higher (lower affinity) than that of the wild type. Truncation of the regulatory element dramatically increased the determined Km (lowered affinity) relative to all three other forms. The activities of the R206H mutant and Q207D variant relative to the wild type were higher, as seen before in assays of FKBP12 inhibition (*cf.* Figure 2A); however, unexpectedly, the truncated form showed by far the highest activity of all. If solely due to autophosphorylation, based on the results of native gel assays, the activity would have been expected to be barely detectable. Hence, the high activity in the ADP-Glo assay, which measures ATP consumption by the amount of ADP produced, was indicative of ATPase activity, not phosphorylation of a kinase polypeptide. In other words, the γ-phosphoryl group of ATP substrate appears to have been transferred to water, with hydroxyl as an attacking nucleophile, rather than to a serine residue on the kinase, with the sidechain hydroxyl as a nucleophile.

In conclusion, the HLH element is hypothesized to perform allosteric regulation by acting as a choke, constricting the active site of the wild-type enzyme, opening up slightly in the recurrent mutant and flooding with water when ablated by deletion. The hyperphosphorylation of caseins by the HLH-truncated form becomes mechanistically clearer in this context, consistent with the hypothesis of polypeptide accessibility as the basis for the gain of function. Mildly increased perturbation of the HLH regulatory element by the R206H point mutation in FOP would slightly release the choke, which in vivo would allow for somewhat increased accessibility relative to the wild type to the serine hydroxyls of BMP Smad C-terminal tails within non-canonically (activin) assembled cytoplasmic signaling complexes.

### 3.7. Model of Heterohexameric Signaling Complex in Support of Polypeptide Accessibility as Structural Basis for Gain of Function of R206H ALK2 Mutant Leading to Classic FOP

**Figure 10:**
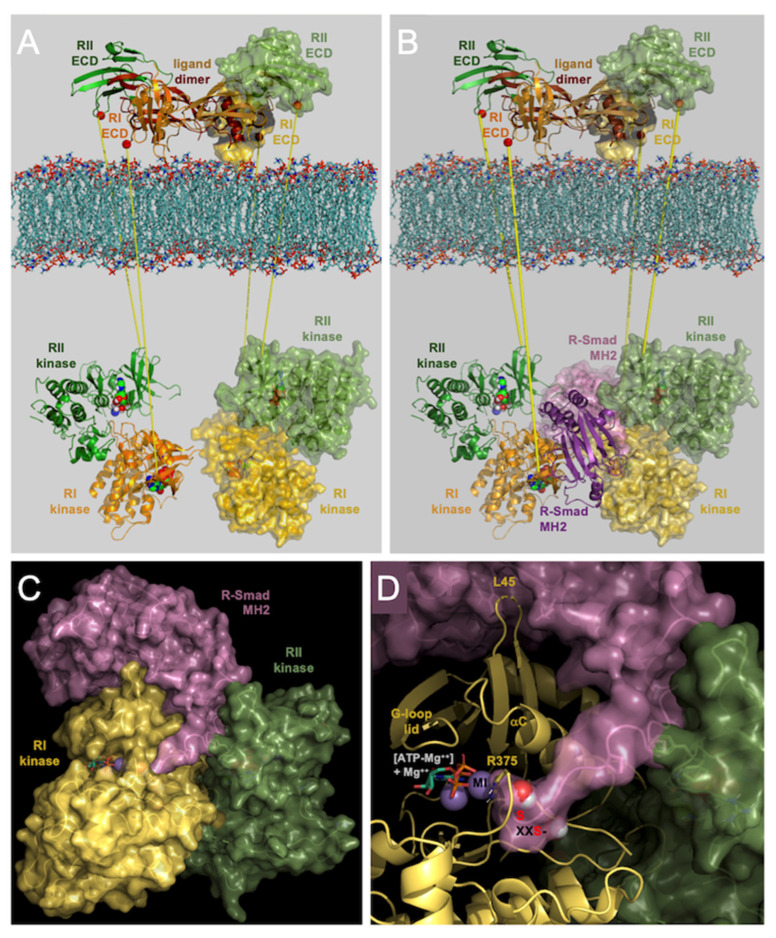
Models of cytoplasmic ALK2-BMPRII-Smad MH2 domain interactions. (**A**) Pre-formed heterodimers of ALK2 and BMPRII kinases. (**B**) Recruitment of two Smad MH2 domains by two kinase heterodimers in juxtaposition. (**C**) One-half of heterohexameric ALK2-BMPRII-Smad MH2 complex depicted in isolation. (**D**) Zoomed view of C-terminal Smad tail situated adjacent to γ-phosphoryl group of metal-ATP complex at the ALK2 active site. Views in c and d are from behind those of b, rotated counterclockwise approximately 60 degrees. Note that the L45 loop of ALK2 is juxtaposed in a crevice to the all-important L3 loop of Smad MH2.

As described in the introduction above, a cytoplasmic BMPRII kinase–ALK2 kinase heterodimer model (Figure 10A, below membrane) was generated and published previously by computational docking via the ClusPro webserver routines [34] that appeared sound not only structurally, but also functionally, since the predicted three-dimensional complex was in keeping with BMPRII-imparted resistance of ALK2 to destabilization by compounds which docked and apparently bound to an allosteric site at the protein–protein interface [20]. Furthermore, a three-dimensional model of the cytoplasmic heterohexameric signaling complex (Figure 10B) was also previously generated by computationally docking Smad MH2 substrate to the BMPRII–ALK2 heterodimer (Figure 10C). Then finally, two BMPRII–ALK2–Smad MH2 ternary complexes were computationally docked to form a heterohexamer, which, due to extensive complementary protein–protein interfaces and interactions, would be anticipated to assemble cooperatively in vivo by ligand mediation [21].

A zoomed view of a ternary complex (Figure 10D) shows the two serines of the C-terminal -S-X--S motif, the sites of activating phosphorylation by ALKs, poised adjacent to the γ-phosphoryl group of ATP that is transferred by the protein kinase. Releasing the choke by mild perturbation of the HLH element is hypothesized to slightly open the active site, allowing for promiscuous entry of the serine hydroxyls in the case of the recurrent FOP mutant, R206H.

Note that for a stable heterohexameric signaling complex to form, a pair of type II kinase–ALK2 heterodimers must be brought into proximity, which in FOP, results from ACVR2A/ActRIIa extracellular domains (ECDs) binding activin A, even without ALK2 ECD binding, which is essentially nonexistent [35]. Such complexes form with wild-type ACVR1/ALK2; however, without the dysregulated allosteric release of the choke, the serine hydroxyls of the C-terminal tails of BMP Smads do not promiscuously enter the active site, or at least to an extent over a threshold to relay signals downstream to trigger heterotopic ossification, hence the disparity observed with activin.

Thus, FOP due to the recurrent R206H mutation is an allosteric disorder, resulting from mild loss of an inhibitory choke at the active site, not diminished binding by an inhibitory protein. Unexpectedly, the second simplest basis postulated initially in 2007—increased affinity for or interaction with the immediate downstream effector, the BMP Smads—appears to be the case; however, this is not due to enhanced binding to the proximal L45 loop specificity determinant [36,37] but occurs indirectly through allosteric propagation of conformational change from the mutationally perturbed HLH element to the polypeptide-interacting perimeter of the active site. Allostery in disease is perhaps one of the most common, widespread mechanisms of dysregulation [38].

## 4. Discussion and Conclusions

Given the juxtaposition between the two sites of interaction, structure-based (TβRI/ALK5; PDB 1B6C) homology modeling indicated that the recurrent FOP mutation led to either a loss of FKBP12 inhibitory protein binding or gain of Smad substrate recruitment [3]. Analyses herein of FKPB12 binding and kinase inhibition in vitro, combined with effects on signaling in primary culture cells, showed that (1) the gain-of-function mutations diminished binding by only a factor of a few fold, (2) FKBP12 acted more like a buffer rather than strict inhibitor and (3) at physiological concentrations of FKBP12, activity of the mutant kinase was suppressed to the level of the unbound wild-type kinase, which did not signal detectably in primary mouse embryonic fibroblasts. Hence, alteration of FKBP12 binding was ruled out as the underlying basis of FOP, consistent with the findings of Hatsell et al. that FK506-mediated abrogation of FKBP12 binding by wild-type ACVR1/ALK2 in vivo was insufficient to account for the gain of function triggering FOP [10].

As mentioned above, altered interaction with the substrate of the kinase, the loosely structured C-terminal tails of BMP Smad-MH2 domains, was concluded to be causative of FOP due to the recurrent FOP mutation. Ironically, enhanced Smad binding at the L45 loop of the folded core proximal to the R206H mutation was ruled out early on as the protein structural—functional basis of the gain of function. Phosphorylation of Smad-MH2 by [γ-^33^P]ATP autoradiography was not detected in assays that yielded robust autophosphorylation or phosphotransfer to caseins, the surrogate substrates. Pre-incubation of ALK2 kinase forms with SUMO-BMPRII fusion protein, which was active with respect to binding by native PAGE, also failed to yield detectable Smad-MH2 phosphorylation. A similar result with ALK2 kinase was obtained by Sapkota and coworkers in their analysis of the differential effects of the inhibitor LDN-193189 through autoradiography [39]. Specifically, ALK2, in comparison with ALK3, ALK4 and ALK5 kinases, produced strong autophosphorylation signals, yet only faint modification of a full-length Smad substrate, which in addition to the DNA-binding MH1 domain included the unstructured linker that may have served as a weak non-specific substrate in place of the C-terminal serine residues of MH2 domains modified in signal transduction.

Although diminished inhibitory protein binding was ruled out as causative of classic FOP, and enhanced Smad interaction initially so, we did explicitly find that alteration of the helix–loop–helix regulatory element, due to the recurrent mutation, the activating aspartate substitution or by total truncation, elevated basal kinase activity as judged particularly by phosphorylation of a casein surrogate. Disruption of the conserved ion pair between the HLH element and the N-lobe of the kinase by the R206H mutation led to a several-fold increase in the basal phosphotransfer activity relative to the wild type. This increase appears not to be due to rearrangement of the active site to increase the catalytic rate, but instead due to increased accessibility of ATP and polypeptide substrates, the latter either surrogate in the case of caseins, or putatively C-terminal BMP Smad MH2 tails in ligand-mediated, cooperatively assembled heterohexameric signaling complexes in vivo.

The proposed mild loss of an inhibitory choke at the active site is consistent with the pathology of FOP. That is, due to the relatively benign effects in utero and the episodic nature of flare-ups of soft tissue, the functional consequences of the mutation must be rather minimal, such as a slight elevation in the activity of the unphosphorylated, autoinhibited enzyme. The level for the mutant is likely sufficiently close to a threshold, which in certain cellular contexts, such as inflamed tissue, allows for localized BMP signaling to extents and for durations that trigger FOP. Since the recurrent mutation is heterozygous and phenotypically dominant, resulting in the dysregulation of half of the ALK2 receptor pool, the modest effect on activity observed in vitro might actually be even weaker in vivo. Thus, of note, the inhibitory activity of a therapeutic need not be overly high or a dosage strategy overly intense to dampen and maintain total basal activity at sub-threshold levels in tissues of FOP patients presenting with classic FOP due to the recurrent R206H mutation in the HLH element.

## Data Availability

Not applicable.
